# A New Broth for the Sick

**Published:** 1855-04

**Authors:** Justus Lieber


### SELECTIONS.

From the Dublin Med. Press.

*A New Broth for the Sick.* By Prof. JUSTUS LIEBER.

To prepare this broth half a pound of the flesh of a recently killed animal (beef, or the flesh of a fowl) is chopped fine, and well mixed with a pound and an eighth of distilled water, to which four drops of pure muriatic acid, and from half to a drachm of common salt, have been added. After an hour, the whole is thrown on a common hair sieve, and the fluid is allowed to run off without pressure. The first portion, which is turbid is poured back, until the fluid runs off clear. On the fleshy residue in the sieve half a pound of distilled water is thrown in small portions. In this way a pound of fluid (cold extract of meat) is obtained, of a red color, and an agreeable taste of broth. The sick are allowed to drink a cupful. cold, at pleasure. It must not be heated, as it then becomes turbid, and deposits a thick coagulum of animal albumen and hematine.

The sickness of a young female servant, from typhus, in my house, gave occasion to this preparation. It was called forth by a a remark of my medical attendant, that, in certain conditions of this disease, the greatest difficulty, which presented itself to the physician, lies in an imperfect digestion—a consequence of the condition of the intestines, and the difficulty of obtaining food suitable for digestion and the formation of blood. Generally, broth prepared by boiling is deficient in all those ingredients of meat which are nesessary for the formation of the albumen of the bl od, and the yoke of an egg, which is added, is very poor in this substance, as it contains, on the whole, 82½ per cent of water and 17½ of egg albumen, or a substance analagous to it, and whether this substance, in its nutritive qualities, is equal to the albumen of flesh, is, according to the investigations of Magendie, at least, doubtful. Besides the flesh albumen, the new broth contains a certain quantity of hematine and therein a large quantity of iron necessary for formation of blood corpuscles, and, lastly, the muriatic acid for digestion. A great hindrance to the employment of this broth in summer is its changeableness in hot weather. It undergoes fermentation, as sugar with yeast, without giving a disagreeable odor. What substance causes this change it is very desirable to ascertain. On that account, the flesh must be treated with very cold water, in a cool place. Ice-water and cooling with ice removes this difficulty. But, above all things, care must be observed that the flesh is used fresh, and not several days old. In the hospitals of Munich, and in private practice, this broth has been employed with great advantage.—*Annales der Chemie and Annals of Pharmacy.*

